# Coumestrol from the national cancer Institute’s natural product library is a novel inhibitor of protein kinase CK2

**DOI:** 10.1186/2050-6511-14-36

**Published:** 2013-07-11

**Authors:** Shu Liu, David Hsieh, Yi-Lin Yang, Zhidong Xu, Csaba Peto, David M Jablons, Liang You

**Affiliations:** 1 Thoracic Oncology Laboratory, Department of Surgery, Helen Diller Family Comprehensive Cancer Center, University of California, 2340 Sutter Street, N-221, San Francisco, CA 94115, USA

**Keywords:** Coumestrol, CK2 inhibitor, Natural product, ATP-competitive

## Abstract

**Background:**

Casein kinase 2 (CK2) is involved in various cellular events such as proliferation, apoptosis, and the cell cycle. CK2 overexpression is associated with multiple human cancers and may therefore be a promising target for cancer therapy. To identity novel classes of inhibitors for CK2, we screened a natural product library obtained from National Cancer Institute.

**Methods:**

The quantitative luminescent kinase assay ADP-Glo™ was used to screen CK2 inhibitors from the natural product library. The same assay was used to determine cell-free dose-dependent response of CK2 inhibitors and conduct a kinetic study. Docking was performed to predict the binding patterns of selected CK2 inhibitors. Western blot analysis was used to evaluate Akt phosphorylation specific to CK2 and apoptosis effect. The cell viability assay CellTiter-Glo® was used to evaluate the inhibition effects of CK2 inhibitors on cancer cells.

**Results:**

We identified coumestrol as a novel reversible ATP competitive CK2 inhibitor with an IC_50_ value of 228 nM. Coumestrol is a plant-derived compound that belongs to the class of phytoestrogens, natural compounds that mimic the biological activity of estrogens. In our study, coumestrol showed high selectivity among 13 kinases. The hydrogen bonds formed between coumestrol and the amino acids in the ATP binding site were first reviewed by a molecular docking study that suggested a possible interaction of coumestrol with the hinge region of ATP site of CK2. In addition, coumestrol inhibited cancer cell growth partially through down-regulation of CK2-specific Akt phosphorylation. Finally, coumestrol exerted strong inhibition effects on the growth of three cancer cell lines.

**Conclusion:**

Our study shows that coumestrol, a novel ATP competitive and cell permeable CK2 inhibitor with submicromolar IC50, had inhibition effects on the growth of three cancer cell lines and may represent a promising class of CK2 inhibitors.

## Background

CK2 (previously referred to as casein kinase II) is a serine/threonine protein kinase composed of 2 catalytic subunits (α and/or α’) and 2 regulatory subunits (β). The alpha and/or alpha’ are linked through two beta subunits to form a stable heterotetrameric structure. CK2 catalyzes the phosphorylation of more than 300 substrates and is itself an evolutionary conserved kinase in eukaryotic cells. Most of the over 300 CK2 substrates have been found to be transcriptional factors (60), effectors of DNA/RNA structure (50) or signalling proteins (more than 80), and a limited number are metabolic enzymes [1]. As such, CK2 plays a critical role on multiple cellular processes, including cell survival [2], apoptosis [3], RNA synthesis [4] and cell transformation [5]. Moreover, CK2 is used by over 40 viruses [1] to phosphorylate the proteins that are essential to their life cycle, including Human Immunodeficiency Virus [6,7], Hepatitis B and C Viruses [8,9], and Human Cytomegalovirus [10].

In addition to its role in viral diseases, CK2 has been reported to be involved in a wide range of neurodegenerative disorders, inflammatory processes, diseases of the vascular system, skeletal muscle, and bone, as well as various types of cancer [11], including adenocarcinoma of the colon [12-14], kidney [15], prostate [16,17] and breast [18,19]. The level of CK2 overexpression in colorectal carcinomas was found to range from 38% to 781% when compared to the corresponding non-neoplastic colorectal mucosa in 20 patients [20]. The average kinase CK2 activity from 21 different renal clear cell carcinomas samples was 610 U/mg compared to 318 U/mg U/mg in the corresponding ipsilateral control tissues [15]. In breast cancer, CK2 levels in seven samples showed CK2 activity increased more than 10-fold compared to control [21]. Therefore, regulating CK2 activity may be a promising therapeutic intervention for cancer [22].

In this study, we screened a natural compound library from the National Cancer Institute (NCI) for potential CK2 inhibitors via a cell-free kinase assay. Through this effort, coumestrol was identified as a novel CK2 inhibitor and was further evaluated for its dose-dependent inhibition effect on CK2 kinase activity in a cell-free manner and in three cancer cell lines.

## Methods

### Cell culture

HeLa, A549 and Jurkat cell lines were purchased from American Type Culture Collection (Manassas, VA). Hela cells were grown in Dulbecco’s modified Eagle’s medium; A549 and Jurkat cells were cultured in RPMI 1640. Both media were supplemented with 10% fetal bovine serum, 10 units/ml penicillin and 10 μg/ml streptomycin at 37°C and 5% CO2.

### Compound library

The Natural Products Set II (NCI, Bethesda, MD) was used to screen novel CK2 inhibitors. This set of 120 compounds was selected out of 140,000 compounds of the Developmental Therapeutics Program Open Repository Collection. Selection criteria for the compounds were based on origin, purity (>90% by ELSD, major peaks show correct mass ion), structural diversity and availability of compound. Each of the two 96-well polypropylene microtiter plates contains 60 compounds with the outside rows and columns of the plate left empty. Plates are stored at −20°C dry. Each well was deposited in 0.20 μM of compound plus 1 μl of glycerol; adding 19 μl of DMSO to each well can produce 20 μl of a 10 mM solution of each compound.

### Cell viability assay

The CellTiter-Glo® luminescent cell viability assay (Promega, Madison, WI) was used to evaluate the cytotoxicity of coumestrol. Cancer cells were seeded in 96-well plates. After 24 hours of attachment to the bottom of the plates, cells were treated by a serial dilution of coumestrol for 72 hours. Then, 50 μl of the CellTiter-Glo reagent was added directly into each well for a 10-minute incubation. The plate was read by GloMax® 96 microplate luminometer (Promega, Madison, WI) after incubation to monitor the luminescence signal generated by the luciferase-catalyzed reaction of luciferin and ATP. A dose–response curve was then plotted as a function of coumestrol concentration used for treatment and luminescence signal.

### Kinase assay

The ADP-Glo™ kinase assay (Promega, Madison, WI) was used to screen two plates of Natural Compound Set II for their CK2 inhibition effects or for a kinase selectivity study. The kinase assay was carried out in a 96-well plate in a volume of 25 μl solvent containing 4 μl of 0.1 μg/l Casein kinase 2 (Millipore, Bedford, MA), 5 μl of 1 mM CK2 substrate peptide HRRRDDD-SDDD-NH2 (Millipore, Bedford, MA), 1 μl of serially diluted coumestrol (Fisher Scientific, Pittsburg, PA), and 15 μl of 10 μM ATP (Promega, Madison, WI). Reactions in each well were started immediately by adding ATP and kept going for half an hour under 30°C in a M-36 microincubator (Taitec Co., Tokyo, Japan). After the plate cooled for 5 minutes at room temperature, 25 μl of ADP-Glo reagent was added into each well to stop the reaction and consume the remaining ADP within 40 minutes. At the end, 50 μl of kinase detection reagent was added into the well and incubated for 1 hour to produce a luminescence signal.

### Molecular docking

A molecular docking study of screened compounds was performed using Discovery Studio (Accelrys, San Diego, CA). One of the crystal structures of CK2/CX-4945 (PDB ID: 3PE1) was chosen to prepare for the receptor and the binding site because of the low resolution of 1.60 Å. Chain A of the crystal complex was defined as the receptor and the site occupied by CX-4945 was defined as the binding site. Sphere of the binding site was added by the program simultaneously. The high throughput virtual screening protocol LibDock was chosen to perform docking. Compounds to be docked were prepared via the PrepareLigand protocol to give 3D coordinates and confirmation. Number of Hotspots generated by LibDock was set as 100 for each case while the remaining parameters were unchanged. After docking, binding poses of compounds were assessed by LibDock Score and visual inspection to identify the correct poses. CK2/ANP (PDB ID: 3NSZ) was then superimposed with modeled complexes. Protein CK2 in the CK2/ANP complex was deleted to leave the ANP overlayed with predicted poses.

### Apoptosis assay

Cells were harvested and stained using an annexin V-FITC apoptosis detection kit according to the manufacturer’s protocol (R&D systems, Minneapolis, MN). Stained cells were analyzed immediately by flow cytometry (FACScan; Becton Dickinson, Franklin Lake, NJ). Early apoptotic cells with exposed phosphatidylserine but intact cell membranes bound to annexin V-FITC, but not to propidium iodide. Cells in necrotic or late apoptotic stages were labeled with both annexin V-FITC and propidium iodide.

### RNA interference

Cells were seeded in a 6-well plate as 50,000 cells/well with fresh media without antibiotics 24 h before transfection, with a target of 30–50% confluency at the time of transfection. CK2a siRNA (ON-TARGET plus SMARTpool) and control siRNA were purchased from Thermo Scientific (Waltham, MA, USA). Cells were transfected with 50 nmol/l of siRNA using Lipofectamine RNAiMAX (Invitrogen, Carlsbad, CA, USA) according to the manufacturer’s protocol. Adequate inhibition of the siRNA-mediated knockdown was confirmed by Western blot. The pcDNA3.1-CK2a or control pcDNA3.1-LacZ plasmid vectors were then transfected into the A549 cells (0.5 lg/ml in 24-well plate) using Lipofectamine 2000 transfection reagent (Invitrogen), following the manufacturer’s protocol. After siRNA transfection, the plates were incubated for 72 hrs at 37°C before further analysis.

### Western blot analysis

After treatment with indicated concentrations of coumestrol or 48 hours, A549 cells were washed with PBS and centrifuged. Cell pellets were lysed with M-PER Mammalian Protein Extraction Reagent (Thermo Scientific) supplied with Complete Protease Inhibitor Cocktails (Roche), and protein concentration was measured with a colorimetric BCA Protein Assay Kit (Pierce). Total protein samples (50 μg) were separated on 4–20% precast polyacrylamide gels (BioRad) and transferred to PVDF membranes. Membranes were blocked with 5% nonfat milk in Tris Buffered Saline-Tween (TBS-T) and incubated with primary antibodies followed by HRP-conjugated secondary antibodies. Immunoreactive proteins were visualized using SuperSignal West Femto Chemiluminescent Substrate (Thermo Scientific). Primary antibodies used were: rabbit anti-Akt1 (4685; Cell Signaling), rabbit anti-PARP (9542; Cell Signaling), rabbit anti-phospho-Akt1 (S129) (ab133458; Abcam) and mouse anti-β-actin (A2228; Sigma).

### Statistical analysis

Data are shown as mean values ± standard deviation (SD). Student’s *t*-test was used to compare cell viability for different treatments. Statistical analysis was carried out using SPSS (version 10.0, Chicago, IL). Significance was defined as p < 0.05 with two-sided analysis. The half maximal inhibitory concentration (IC_50_) values was determined using GraphPad Prism® log (inhibitor) vs. response (variable slope) software (version 6.01, La Jolla, CA).

## Results

### Screening CK2 inhibitors from a natural compound library

**Figure 1:**
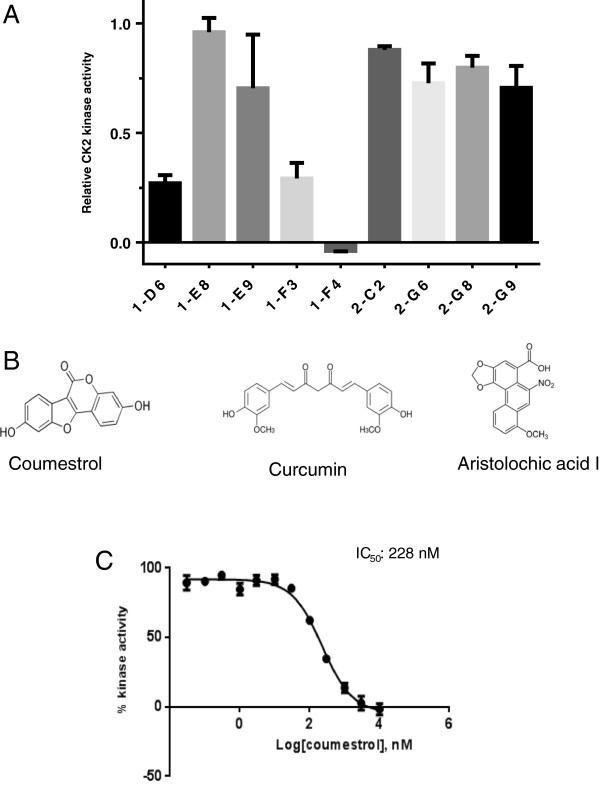
**Inhibition effects of representative natural compounds on CK2 kinase activity. A**. Natural compounds from NCI plates were screened for their CK2 inhibition activities at 10 μM using a kinase assay. Relative kinase activity of CK2 is shown on the Y axis as a relative number to the control that did not have inhibitor treatment. Compounds 1-F4 (coumestrol), 1-D6 (curcumin) and 1-F3 (aristolochic acid I) showed more than 50% inhibition on kinase activity at 10 μM. Data represents the average of duplicates and bars indicate standard deviation. 1-X represents compounds from NCI plate 13091250 and 2-X represents compounds from NCI plate 13091251. **B**. Chemical structures of coumestrol, curcumin and aristolochic acid I. **C**. IC_50_ of coumestrol.

Library screening showed that three compounds had various levels of inhibitory effects on CK2 activity when compared to control samples (DMSO) (Figure 1A). These effects were evaluated in the presence of 10 μM ATP. The three compounds were coumestrol (1-F4; 3,9-Dihydroxy-6-benzofurano [3,2-c]chromenone), curcumin (1-D6; (1*E*,6*E*)-1,7-Bis(4-hydroxy-3-methoxy phenyl)-1,6-heptadiene-3,5-dione) and aristolochic acid I (1-F3; 8-methoxy-6-nitro phenanthro[3,4-*d*] [1,3] dioxole-5-carboxylic acid (Figure 1B). Although all curcumin and aristolochic acid 1 showed more than 50% inhibition of kinase activity at 10 μM, coumestrol completely inhibited CK2. Therefore, the inhibition effect of coumestrol was further elucidated by kinase assay and a dose-dependent response of coumestrol was plotted to yielded an IC_50_ of 228 nM against CK2 (Figure 1C).

### Coumestrol selectively inhibits CK2 kinase activity

To further elucidate the specificity of coumestrol to CK2, we tested a group of 13 kinases representing nine kinase families. We found that coumestrol showed no inhibitory effects on eight kinases: CK1, HER2, MAP2K, MET, AKT2, SRC, PAK1 and mTOR at 10 μM in the presence of 10 μM ATP (Table 1). In contrast, coumestrol inhibited 100% of the kinase activity of CK2, 36% of DYRK1a, 40% of GSK3b and 47% of JAK2. Although coumestrol had a relatively high inhibition of 59% against vascular endothelial growth factor receptor 3 (VEGFR3), this inhibition might be compromised because VEGFR3 is a proven drug target [23]. Overall, our results suggest that coumestrol selectively inhibits kinase activity of CK2 in a cell-free manner.

**Table 1:** Specificity spectrum of coumestrol

**Protein kinase**	**Kinase activity %**
CK2	0
CK1	119
DYRK1a	64
HER2	98
MAP2K	139
MET	101
VEGFR	41
AKT2	164
GSK3b	60
SRC	166
PAK1	129
mTOR	110
JAK2	53

Remaining kinase activity was determined in the presence of 10 μM coumestrol and 10 μM ATP and expressed as a percentage of the control without inhibitor. *CK2*, Casein kinase 2; *CK1*, Casein kinase 1; *DYRK*, Dual-specificity tyrosine-(Y)-phosphorylation regulated kinase; *HER2*, Human epidermal growth factor receptor 2; *MAP2K*, Mitogen-activated protein kinase kinase; *c-Met*, c-Met receptor tyrosine kinase; *VEGFR3*, Vascular endothelial growth factor receptor 3; AKT, also known as protein kinase B (PKB); *GSK*, Glycogen synthase kinase; *SRC*, *src* Gene encoded non-receptor tyrosine kinase; *PAK1*, p21 Protein (Cdc42/Rac)-activated kinase 1; *mTOR*, Mammalian target of rapamycin; *JAK*, Janus kinase.

### Coumestrol reversibly inhibits CK2 kinase activity as an ATP competitor

**Figure 2:**
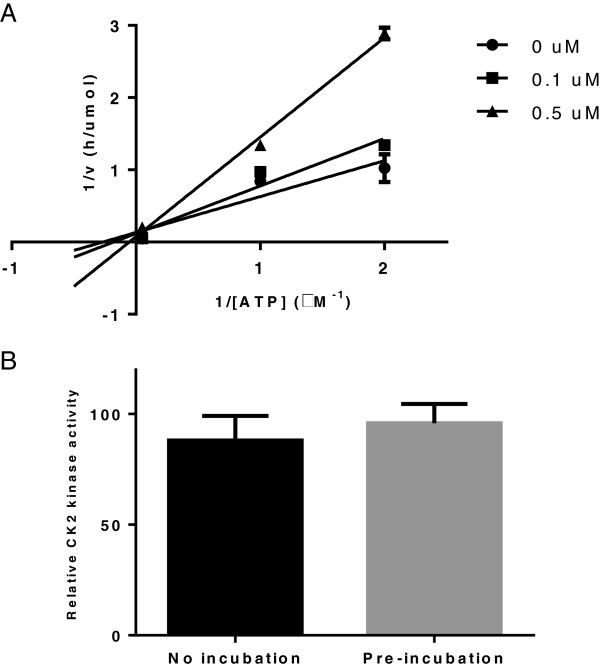
**Kinetic analysis and reversibility assay of CK2 inhibition by coumestrol. A**. Lineweaver-Burk plots of inhibition of CK2 by coumestrol: ● 0 μM, ■ 0.1 μM, ▲ 0.5 uM. Substrate concentration was fixed at 200 μM. The plots illustrate that coumestrol is an ATP competitive CK2 inhibitor. The data represents means of duplicate experiments. **B**. Reversibility study of coumestrol. Coumestrol was pre-incubated with 100 ug/ml of CK2 for 1 hour and then a kinase assay was performed at a final concentration of coumestrol at 100 uM as described in Methods. CK2 kinase activity is represented as relative CK2 activity to control. Data points represent the average of triplicate experiments and bars indicate standard deviation.

Having shown the affinity and selectivity of coumestrol as a CK2 inhibitor, we next sought to determine the inhibition mode of coumestrol by using a kinetic study. The resulting Linewear-Burk plots showed that coumestrol is an ATP competitor (Figure 2A). A reversibility study was then performed in which coumestrol was pre-incubated with CK2 in a concentration of 100 μg/ml for one hour. A CK2 kinase assay was then done with the final concentration of 100 μM coumestrol. Pre-incubation did not affect the amount of kinase activity (Figure 2B), indicating that coumestrol is a reversible inhibitor toward CK2.

### Coumestrol forms hydrogen-bond interactions with the hinge region of the ATP site of CK2

**Figure 3:**
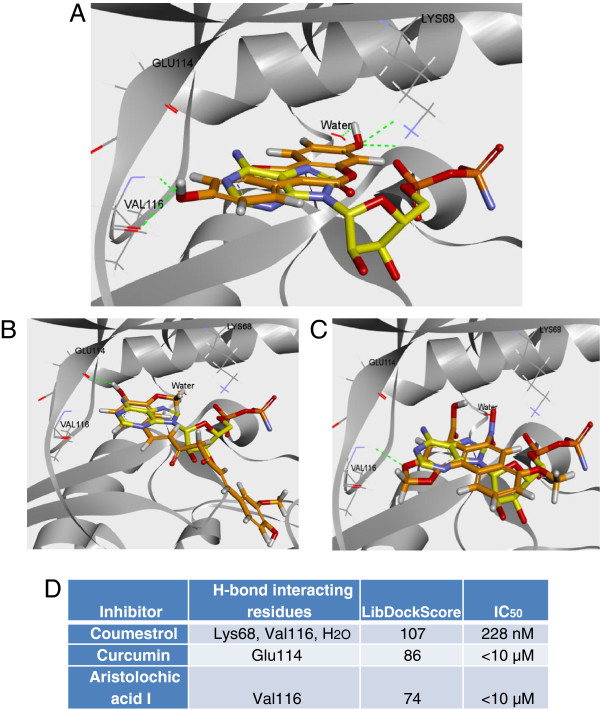
**Predicted binding of coumestrol, curcumin and aristolochic acid I in the ATP binding site of CK2.** The binding mode of coumestrol **(A)**, curcumin **(B)**, and aristolochic acid 1 **(C)** in the active site of CK2 was predicted by docking. The three compounds (carbon atoms colored in orange) and an ATP analog, phosphoaminophosphonic acid-adenylate ester (carbon atoms colored in yellow), were overlayed together. The docked pose indicated that hydrogen bonds were formed between coumestrol and CK2. Hydrogen bonds are labeled in green dotted lines. CK2 residues adjacent to coumestrol Glu114, Val116, Lys68, and a conserved water molecule, are shown in line representation along with coumestrol (in A), curcumin (in B), or aristolochic acid 1 (in C) (red represents oxygen, blue represents nitrogen and white represents hydrogen). The rest of the CK2 protein is shown in the flat ribbon. **(D)**. Summary of interactions of coumestrol, curcumin and aristolochic acid I with CK2. Residues that make H-bonds, LibDockScore and IC_50_ values of the three inhibitors were listed.

We next examined the binding pattern of coumestrol as an ATP competitor via docking. For this experiment, coumestrol and two other compounds identified from screening—curcumin and aristolochic acid I—were docked into the ATP site, although the inhibition modes of the latter two were not determined. The results suggested that coumestrol forms hydrogen-bond interaction with the hinge region residue Val 116 (Figure 3A). In addition, coumestrol formed another H-bond with Lys68 and a conserved water molecule inside the ATP binding site (Figure 3A). Curcumin and aristolochic acid I were successfully docked into the ATP site as well, suggesting they might be ATP competitors (Figure 3B, 3C). Curcumin established an H-bond with Glu 114 and aristolochic acid I with Val 116. LibDockScores of the three inhibitors correlated with their single concentration inhibition at 10 μM against CK2 (Figure 3D). The docking result for coumestrol provides possible interaction patterns of the compound with CK2. It also suggests that curcumin and aristolochic acid I are potential CK2 inhibitors that regulate kinase activity through competition with ATP; however, proving the inhibition mode would require kinetic studies.

### Coumestrol inhibits CK2 kinase activity *cell*-*free* and downstream Akt phosphorylation in A549 lung cancer cells

**Figure 4:**
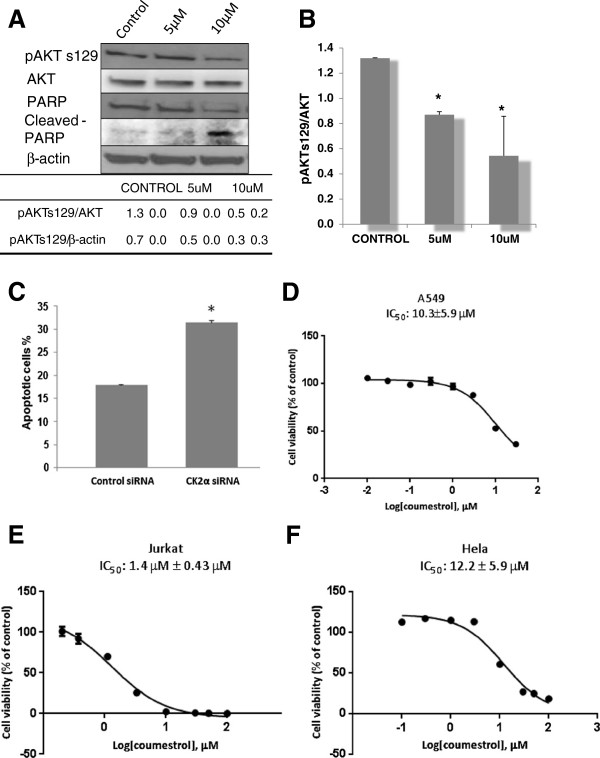
**Downstream signalling in A549 lung cancer cells treated with coumestrol and inhibition effects of coumestrol on cellular viability in three cancer cell lines. A**. Phosphorylated Akt (Ser129), total Akt, and PARP were measured by western blot analysis. B-actin was used as loading control. Expression of pAKT s129 was quantified using ImageJ software and the mean of relative expression level to β-actin or to total AKT was presented (mean ± SD). **B**. Coumestrol significantly decreased the expression of pAKT s129 in A549 cells (*, p < 0.05, Student t-test). **C**. Annexin V analysis of apoptosis induced by CK2α siRNA. A549 cancer cells were treated with 100 nM CK2α siRNA and 100 nM control siRNA for 72 h. **D**, **E**, **F**. A549, Jurkat and Hela cells were cultured in the absence and in increasing concentrations of coumestrol (0.1 uM to 100 μM) as indicated. Cellular viability (normalized to DMSO control) was measured after 48 hours using CellTiter-Glo®Luminescent Cell Viability Assay. Data points represent the average of IC_50_ value of coumestrol in triplet experiments and bars indicate SD.

Since CK2 showed a dose-dependent response to coumestrol inhibition cell-free, we examined the inhibition effects of coumestrol on intact cancer cells. A549 lung cancer cells were treated with either 5 μM or 10 μM coumestrol for 48 hours. Interestingly, Akt Ser129, which is phosphorylated by CK2, also showed significantly decreased phosphorylation in A549 cells (Figure 4A). However, total CK2, total Akt and β-actin were comparable. Quantification of expression of pAKT s129 compared to total AKT using different doses of coumestrol in A549 cells showed that coumestrol significantly decreased the expression of pAKT s129 (Figure 4B). Increased cleaved poly ADP-ribose polymerase was also detected in cell lysate treated with 10 uM of coumestrol (Figure 4A), indicating increased caspase-dependent apoptosis of cancer cells after coumestrol treatment. A549 cancer cells were also treated with CK2α siRNA to analyze induced apoptosis. The percentage of apoptotic cells treated with CK2α siRNA was significantly increased, demonstrating a correlation between reduced cell viability and CK2 activity (Figure 4C).

### Coumestrol exerts inhibition effects on growth of cancer cells

Finally, we compared the inhibition effects of coumestrol on three cancer cell lines. A549, Jurkat and Hela cells were treated with serially diluted coumestrol for 72 hours, and cell viability was measured via the CellTiter-Glo luminescent cell viability assay. From the dose response curve, IC_50_ values were calculated in A549 (10.3 ±5.9 μM) Jurkat (1.4 uM ± 0.43), and Hela (12.2 ± 5.9 μM) cancer cells (Figure 4D,E,F). The results indicate that coumestrol shows strong inhibition effects towards Jurkat, A549 and Hela cells.

## Discussion

Historically, natural products are important starting materials in the lead discovery phase of the drug discovery process and have been a major source for new chemical entities [24]. More recently, combinatorial chemistry has become an alternative choice. However, the number of lead optimization candidates yielded by combinatorial chemistry has been much less than expected [25]. The underlying reason might be that chemical structures obtained through combinatorial approaches lack essential lead-like properties [24]. Because of these problems, and the fact that CK2 overexpression is associated with multiple human cancers and may therefore be a promising target for cancer therapy, we decided to screen the natural product library obtained from the NCI to identify novel CK2 inhibitors. For this purpose, we used a cell-free kinase assay to screen the libraries. Coumestrol was identified as a promising CK2 inhibitor. Kinetic assays in our study also showed that coumestrol is an ATP competitive and reversible inhibitor toward CK2. The results, combined with those from a kinetic study, led us to identify and validate coumestrol as a novel CK2 kinase inhibitor.

To the best of our knowledge, our study is the first to show that coumestrol is a CK2 kinase inhibitor in both cell-free assay and cancer cells. The cell-free IC_50_ value of coumestrol (0.23 μM) on CK2 kinase activity is comparable to that of several well established CK2 inhibitors, such as 2-Dimethylamino-4,5,6,7-tetrabromo-1*H*-benzimidazole (DMAT) (0.15 μM), [5-oxo-5, 6-dihydroindolo-(1, 2-a) quinazolin-7-yl] acetic acid (IQA) (0.39 μM), 4,5,6,7-tetrabromo benzotriazole (TBB) (0.50 μM) [22] and 1, 3, 8-trihydroxyanthraquinone (emodin) (0.89 μM) [26].

We also showed that coumestrol triggered apoptosis in cancer cells. Previous studies suggest that CK2 plays an essential role in suppressing apoptosis. Overexpression of CK2 in cancer cells protects cells from etoposide- and diethylstilbestrol-induced apoptosis [27], resulting in suppressed apoptosis mediated through tumor necrotic factor-alpha (TNF-α), TRAIL and Fas L, and augments apoptosis in cells sensitive to these ligands [28]. Treatment of a variety of cancer cells with cell-permeable CK2 inhibitors such as TBB, IQA and DMAT has been shown to induce activation of caspases and then apoptosis [22,29,30]. In our study, coumestrol inhibited Akt/PKB Ser129 phosphorylation in cancer cells. Akt/PKB is activated by CK2 and ensures cell survival via activation of anti-apoptotic pathways, including the NF-κB pathway and suppression of caspase activities [31-33]. Thus, coumestrol induces apoptosis in cancer cells at least partially by inhibiting the Akt/PKB pathway by down regulation of CK2 kinase and then decreased phosphorylation of Akt/PKB Ser129.

Coumestrol belongs to the class of phytoestrogens that includes isoflavones and coumestans. It is the most prevalent derivative of coumestan [34], which can be found in leguminous plants serving as food sources for humans. Coumestrol intake in the Asian population is 10 times greater than that of the non-Asian population [35]. The half-lives of plasma genistein and daidzein, compounds from the same family of coumestrol, were found to be 8.36 and 5.79 hr, respectively, in humans [36]. A pharmacokinetic study of soy-derived phytoestrogens in rats suggested that genestein has a half-life of 4.3 hr, daidzein 2.3 hr and coumestrol 5.5 hr, almost equal to 5.6 hr observed for zearalenone [37].

A specific dietary supplement, selected vegetables (SV), which contains coumestrol, was studied in tumor-bearing mice and in stage IIIB and IV non-small cell lung cancer patients [37]. The study found 53-74% inhibition of tumor growth in mice, but more strikingly, patients in stage IIIB and IV NSCLC who took SV daily for 2–46 months had prolonged survival and attenuation of the normal pattern of progression compared to patients not taking SV [38].

Soy isoflavones, because they are estrogen-like compounds, are thought to have potential side effects on patients with ER-positive breast cancer. The structure of coumestrol is similar to that of estradiol, and coumestrol reportedly can bind to two estrogen receptor subtypes (ERα and ERβ) but with lower binding affinity than that of estradiol [39]. Despite the structural similarities, soy isoflavones bind to ER differently than estrodiol does and are thought to exhibit only beneficial effects of estrogen [40-43]. High consumption of soy foods may reduce the risk of breast cancer [44]. However, whether the use of coumestrol as a cancer treatment may have side effects related to estrogen receptors requires further study.

Coumestrol is a relatively small molecule (MW 268), which provides room for physical/chemical activity modifications. Tumors that overexpress CK2 could be potentially treated with coumestrol or coumestrol derivatives that have better drug-like properties [15,21,45]. Coumestrol and its derivatives can also potentially target several key signaling pathways such as the Akt pathway, a particular example being EGFR mutations [46,47]. Thus, coumestrol may represent a new class of targeted treatments for cancer.

## Conclusions

In this study, we showed that coumestrol is a novel ATP competitive and reversible CK2 inhibitor. Coumestrol not only showed inhibition effects on CK2 (IC_50_ 228 nM) cell-free, but also showed the same effects on CK2 *in vitro*. In addition, coumestrol inhibited the growth of three cancer cell lines, indicating its cell-permeable property. A molecular docking study suggested a possible interaction of coumestrol with the hinge region of ATP site of CK2. Taken together, our findings indicate this compound may represent a promising class of CK2 inhibitors.

## Abbreviations

CK2: Casein kinase 2; SD: Standard deviation; VEGFR: Vascular endothelial growth factor receptor; DMAT: 2-Dimethylamino-4,5,6,7-tetrabromo-1H-benzimidazole; IQA: [5-oxo-5, 6-Dihydroindolo-(1, 2-a) quinazolin-7-yl] acetic acid; TBB: 4,5,6,7-Tetrabromo benzotriazole; Emodin: 1, 3, 8-Trihydroxyanthraquinone

## Competing interests

The authors declare that they have no competing interests.

## Authors’ contributions

SL carried out kinase assay, cell viability assay, molecular docking study, and also drafted the manuscript. DH performed kinase assay and cell viability assay. YLY carried out western blot analysis. ZX carried out the RNA interference, apoptosis and cell line experiments. CP revised the manuscript. SL and LY designed the study and revised the manuscript. All the authors analyzed the results. All authors read and approved the final manuscript.

## Pre-publication history

The pre-publication history for this paper can be accessed here:

http://www.biomedcentral.com/2050-6511/14/36/prepub
